# Oxygen and blood flow: players in the pathogenesis of glaucoma

**Published:** 2008-01-31

**Authors:** Maneli Mozaffarieh, Matthias C. Grieshaber, Josef Flammer

**Affiliations:** University Eye Clinic, Basel, Switzerland

## Abstract

**Purpose:**

The increase of IOP in POAG is due an increased resistance of aqueous outflow through the trabecular meshwork (TM).

**Methods:**

The exact mechanisms leading to the corresponding changes in the TM are not yet known. We know, however, that all risk factors for arteriosclerosis are also risk factors for an increase in IOP.

**Results:**

The association between IOP increase and these factors is relatively weak but nevertheless significant. Similar to the pathogenesis of arteriosclerosis, oxidative stress plays a role in the development of TM damage.

**Conclusions:**

Even less is known about the pathogenesis of glaucomatous optic neuropathy (GON). Obviously the risk factors for arteriosclerosis play a role via increasing the IOP. When corrected for IOP, however, these factors only play a minor role. In contrast, factors associated with disturbed autoregulation, in particular a systemic primary vascular dysregulation (PVD), increase the risk for GON. This is best observed in normal tension glaucoma patients. An insufficient autoregulation increases the chance for an unstable ocular perfusion and thereby an unstable oxygen supply. This, in turn, leads to oxidative stress. The concentration of superoxide (O_2_^-^) within the axons of the optic nerve head increases. If neighboring astrocytes are activated, either by mechanical or by ischemic stress, in excess produced nitric oxide (NO) molecules diffuse also into the axons and fuse with oxygen. The resulting peroxynitrat (ONOO^-^) diffuses within the axons towards the retina and the lateral geniculate nucleus and induces apoptosis.

## Introduction

**Figure 1:**
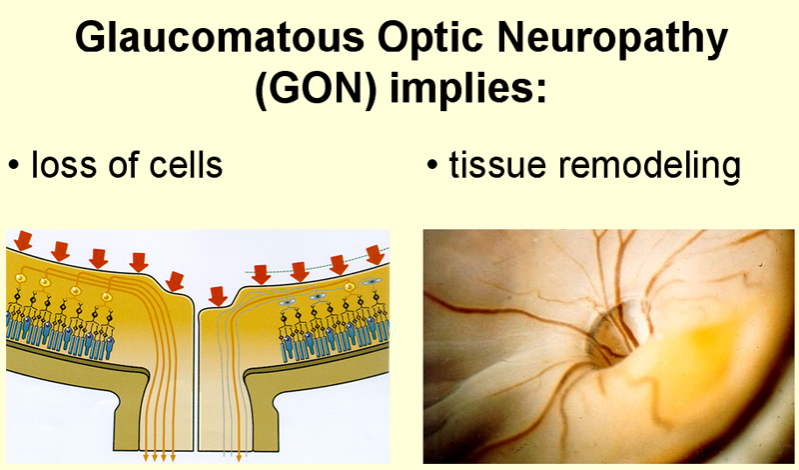
Phenomenology of glaucomatous optic neuropathy. Glaucoma, a progressive optic neuropathy, is characterized by the loss of retinal ganglions cells and their axons and tissue remodelling involving both the optic nerve head and the retina. This leads clinically to a visible cupping of the disc and measurable thinning of the nerve fiber layer of the retina. This figure is reprinted from Flammer J. Glaucoma, Glaucoma A Guide for Patients. An Introduction for Care-Providers. A Quick Reference. 3rd ed. Cambridge: Hogrefe & Huber; 2006. Figure S1.54; p 235.

Glaucoma, a progressive optic neuropathy, is characterized by the loss of retinal ganglions cells and their axons and tissue remodelling involving both the optic nerve head and the retina. This leads clinically to a visible cupping of the disc and measurable thinning of the nerve fiber layer of the retina (Figure 1). The patients experience progressive visual field damage along with a decrease in contrast and color sensitivity [1]. Increased intraocular pressure (IOP) is a major risk factor for primary open angle glaucoma (POAG). Still, the focus on IOP as the only risk factor leaves various questions open. Why don't all patients with glaucomatous damage have an elevated IOP? Why do a majority of people with increased IOP not develop glaucomatous optic neuropathy (GON)? Why does the therapeutic reduction of IOP, although on average improving the prognosis of GON, not stop progression in all patients? Why do some patients require very low, almost non-physiological, IOP levels to stop progression? These observations challenge the pathophysiological concept of glaucoma based solely on IOP. Today, we know that other concomitant factors such as blood flow alterations [2], and oxidative stress [3] play an important role.

## Methods

**Figure 2:**
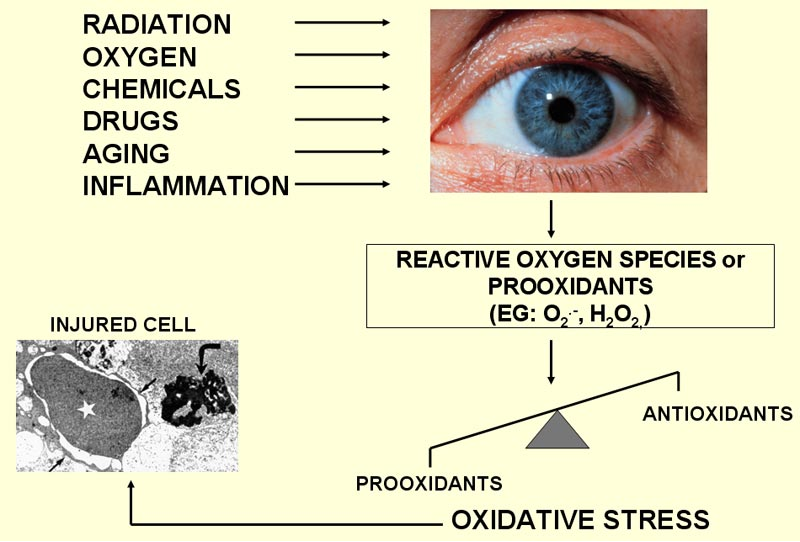
Risks and consequences of oxidative stress. The eye is an organ that is predisposed to great levels of oxidative stress. The eye is constantly exposed to factors such as radiation, chemicals, oxygen, drugs, which induce the formation of reactive oxygen species (ROS) that can ultimately damage cells. This figure is modified from Flammer J. Glaucoma, Glaucoma A Guide for Patients. An Introduction for Care-Providers. A Quick Reference. 3rd ed. Cambridge: Hogrefe & Huber; 2006. Figure S1.29; p 222.

Oxygen is the primary oxidant in metabolic reactions designed to deliver energy from the oxidation of a variety of organic molecules. Metabolism of oxygen by cells generates as a by-product potentially deleterious reactive oxygen species (ROS) [4] (Figure 2). Under optimal conditions the rate and magnitude of oxidant formation is balanced by the rate of oxidant elimination through the action of antioxidants. But even in healthy individuals excess of oxidants may cause some macromolecular damage. An imbalance between prooxidants and antioxidants, in favour of the former, however, results in oxidative stress. This, in turn, may lead to damage of a variety of macromolecules, such as proteins, lipids, sugar residues, or DNA, and thereby leads, in extreme cases, to growth arrest, modulation in form and function and even to death of cells.

## Results

A more or less constant reduction of OBF within a certain limit can be tolerated by the eye. Patients, for example, with severe arteriosclerosis or with reduced OBF due to high level of endothelin (e.g., due to autoimmune diseases) often have a normal or slightly pale optic nerve head, which however is not excavated. This indicates that tissues can actually adapt to lower levels of oxygen. If OBF, however, is markedly reduced an infarction will result. On the other hand, if oxygen supply is unstable (due to fluctuation of perfusion pressure, especially when auto-regulation is defective), oxidative stress results. In other words a constant reduction of OBF (e.g., due to atherosclerosis) is a risk factor for infarction; unstable OBF, however, is a risk factor for GON.

This instability in blood flow leads to a repeated mild reperfusion and thereby to the formation of ROS which damage the mitochondria of the axons especially the mitochondria of the optic nerve head (so called reperfusion injury).

This review summarizes the current evidence supporting the role of ocular blood flow (OBF) and oxidative stress with particular reference to interrelationships between blood flow dynamics and oxidative stress in the pathogenesis of glaucomatous damage.

## Discussion

### The oxygen molecule

#### Ground state oxygen

The oxygen molecule is thought of as the elixir of life, and this benign image is well deserved as long as the molecule remains in its electronic ground state. This molecule when in an electronically excited state, forming reactive oxygen species, becomes delirious, in particular if in excess of cellular antioxidant balance. The oxygen molecule in ground state (the most stable state of oxygen), is a di-radical [5].

A free radical is an atom or a molecule with one or more unpaired electrons in its outer shell; by gaining or loosing an electron these molecules accommodate a much more stable electronic configuration.

**Figure 3:**
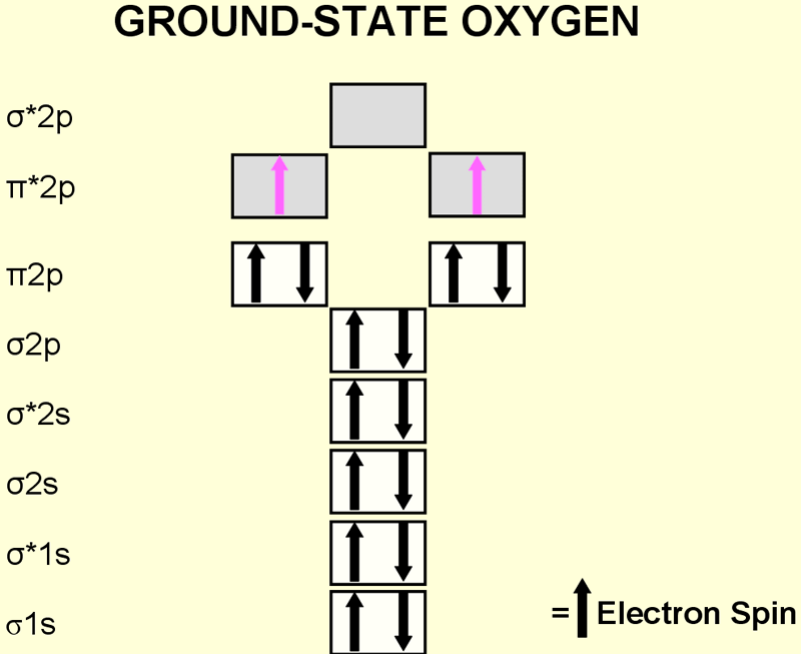
Electron configuration of the diatomic oxygen molecule. Atomic oxygen (atomic number 8) has a total of eight electrons. The oxygen molecule (O^2^) has 16 electrons. In ground state (the most stable state of oxygen), the last two electrons of the oxygen molecule are located in a different π* antibonding orbital. These two unpaired electrons have the same quantum spin number (they have parallel spins) and qualify ground state oxygen to be a di radical.

The question therefore arises as to why the oxygen molecule, a di-radical in ground state, is minimally reactive. In ground state the oxygen molecule has two unpaired electrons, each of which are located in a different pi* antibonding orbital (Figure 3). These two electrons rotate about their own axis in parallel spins. When two free radicals fuse to an new molecule, the electrons of the two molecules that will pair together have different (anti-parallel) spin. Oxygen molecule would need another molecule with two electrons with the same spin, anti-parallel to the spin of the electrons of the oxygen molecule. This however is very rare. Due to this “spin restriction” molecular oxygen in its ground state is minimally reactive.

#### Activation of oxygen

**Figure 4:**
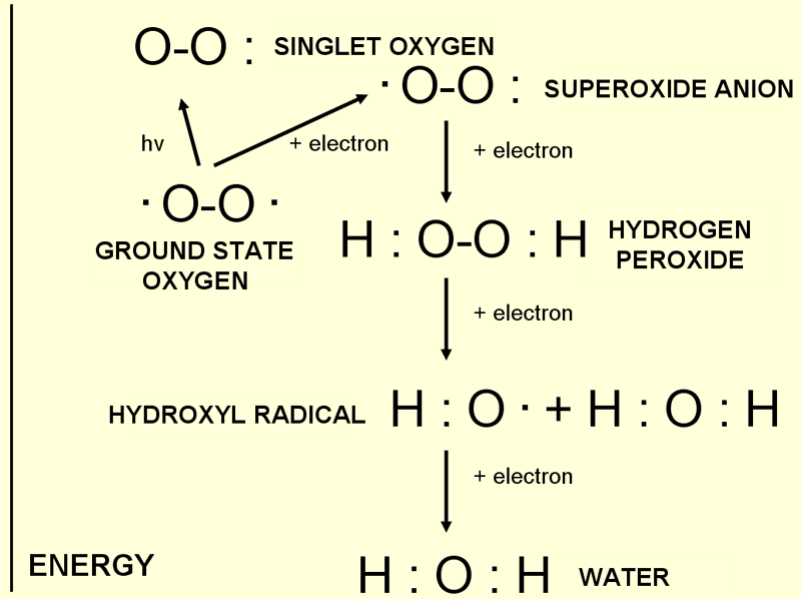
Different energy levels of oxygen and oxygen compounds. Activation of oxygen occurs either through absorption of sufficient energy or through monovalent reduction.

Oxygen can be activated by two different mechanisms. Either, through the absorption of sufficient energy to reverse the spin on one of the unpaired electrons, or through monovalent reduction (Figure 4). If ground state oxygen absorbs sufficient energy to reverse the spin of one of its unpaired electrons, the two unpaired electrons now have opposite spins. This activated form of oxygen, known as singlet oxygen (^1^O_2_) is much more reactive than ground state oxygen and reacts destructively with molecules. The second mechanism of activation is by monovalent reduction [4]. Hydrogen peroxide is a molecule with a high oxidizing capacity. In the presence of reduced transition metals (e.g., ferrous or cuprous ions) hydrogen peroxide can be converted into the highly reactive hydroxyl radical (OH) this reaction is known as the Fenton reaction.

#### Oxidative stress

**Figure 5:**
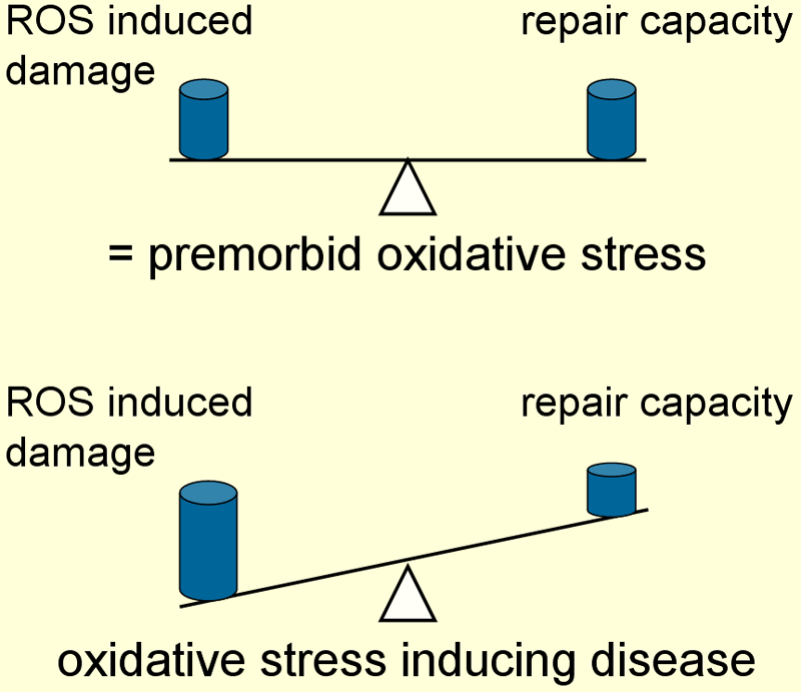
Oxidative stress and disease. In the physiological state, the production of prooxidants is approximately balanced by the antioxidant defence system. The term oxidative stress is referred to when the intensity of prooxidants exceed the antioxidant capacity.

The eye is a unique organ because of its constant exposure to light, atmospheric oxygen, environmental chemicals and physical abrasion. As previously mentioned, metabolism of oxygen by cells generates potentially deleterious reactive oxygen species (ROS). Under optimal conditions the rate and magnitude of oxidant formation is balanced by the rate of oxidant elimination through the action of antioxidants [6]. An antioxidant is by definition any substance that significantly delays or prevents oxidation of a substrate. In very simple words, an antioxidant can neutralize prooxidants. Nature has therefore provided us with mechanisms to help us cope with prooxidants. If ROS production exceeds this capacity, however, oxidative stress damages different molecules. As long as nature is capable of repairing damaged molecules (e.g., DNA) or eliminating damaged molecules (proteins via proteosomes) no major structural damage occurs. But if oxidative stress induced-damage exceeds the capacity of repair mechanisms, structural damage sums up and leads, finally, to a clinically relevant damage that we call a disease, such as glaucoma (Figure 5).

#### Ocular blood flow in glaucoma

The regulation of blood flow in the eye, is different in different tissues. The regulation of retinal blood flow is very similar to the regulation of blood flow in the brain, with the exception that retinal vessels have no autonomic innervation and therefore its regulation depends even more on the activity of endothelium cells. These cells release a number of factors, the so-called endothelium derived vasoactive factors (EDVFs) [7], which on one hand regulate the size of the vessels by influencing vascular smooth muscle cells locally, and on the other hand, via intraluminal release of these factors lead to changes in blood rheology (e.g., by influencing platelet aggregation) and influence the size of the vessels globally. In addition, both the neural and glial cells, also influences the size of the vessels. This is known as neurovascular coupling. If flickering light for e.g., hits the eyes, the vessels dilate within seconds [8]. The exact mechanism of this regulation is not yet known. It is known, however, that the production of nitric oxide (NO) is involved.

The regulation of blood flow of the choroid is very different from that of retinal blood flow [2]. The choroidal vessels are extensively autonomically innervated and the capillaries are fenestrated. Besides providing oxygen and other molecules to the retina, the choroid regulates the temperature of the back of the eye and most probably contributes to the fine tuning of accommodation by regulation of volume. The regulation of temperature can be demonstrated, for e.g., with the observation that when moving from a warm to a cold environment, the choroidal blood flow increases within seconds. If we put one drop of local anesthetics in one eye, but not in the other, the eye that received the local anesthetic drop will adapt much less. The cold response is mediated by cold receptors in the sclera.

Blood flow in the optic nerve head is regulated somewhat similarly to that of the retina with the important exception that no efficient blood brain barrier exists in the optic nerve head [9]. As a consequence circulating molecules such as vasoactive hormones, enzymes, or even drugs, get access to the smooth muscle cells and pericytes of the vessels in the optic nerve head.

On average, blood flow is reduced in glaucoma patients in various tissues of the eye, including the iris, retina, optic nerve and choroids [2,10,11]. Blood flow reduction is even more pronounced in normal tension glaucoma than in high tension glaucoma. Interestingly, reduction in blood flow can also be observed in the nailfold capillaries of fingers of glaucoma patients, indicating that this reduction is not only due to an increase in IOP or glaucomatous damage. There must be a primary component of blood flow dysfunction and this, in turn, is due to a global vascular dysregulation.

### Vascular dysregulation syndrome

#### What is vascular dysregulation?

In biology any cell but also any organ or any living organism must constantly adapt to different environments. This is the reason why only organisms with a good regulation survived evolution. For regulation of any parameter several systems are involved in a way that dysfunctioning of any one of the systems would lead to compensation by another. Furthermore, the regulations of different parameters are often interrelated. Blood flow, for example, is involved in the regulation of oxygen supply, transport of temperature and volume regulation. This explains why a basic dysregulation, such as a vascular dysregulation, leads to many different and seemingly unrelated symptoms.

The vascular system is highly regulated on different levels such as on large or small arteries, capillaries and veins. A dysregulation simply means that this local adaption is not according to the need of the body or its corresponding tissues. This may have local reasons, such as damage of the endothelium cells by rupture of an atherosclerotic plaque. An inflammation of the vessel walls also hinders the local adaption. In this context, however, we are going to discuss the more systemic types of dysregulation. These can be grouped in a primary vascular dysregulation (PVD) and a secondary vascular dysregulation (SVD). In the context of glaucoma, PVD is more important as we shall see later on in this review. We will therefore just summarize some of the aspects of SVD and then describe PVD in more

#### Secondary vascular dysregulation

A secondary dysregulation is due to other diseases; quite often, autoimmune diseases. Patients with a secondary vascular dysregulation normally have high level of circulating Endothelin. The high level of Endothelin leads to a reduction of blood flow both in the choroid and the optic nerve head [12-14]. It does not, however, interfere with autoregulation which is why secondary vascular dysregulation remains a minor risk factor for glaucomatous damage.

As mentioned before the endothelial cells release vasoactive substances not only abluminally, to regulate local resistance, but also intraluminally to induce an overall effect. This effect is very different from one organ to the other due to the fact these substances, while having access to endothelial cells in all organs, have direct access to smooth muscle cells and pericytes only in organs with fenestrated capillaries such as the choroid and thereby also indirectly to the optic nerve head.

Physiologically Endothelin is produced by endothelial cells. Under pathological conditions, however, any cell under marked stress can produce Endothelin and thereby contribute to the level of endothelin in the circulating blood [15-19]. For example, in patients with arthritis the synovial cells produce Endothelin, in multiple sclerosis the lymphocytes and finally in AIDS the macrophages etc.

#### Primary vascular dysregulation

Subjects with a primary vascular dysregulation (PVD) syndrome have an inborn tendency to respond differently to various stimuli such as cold, mechanical or emotional stress and others [20]. Affected subjects also exhibit differences for e.g., in sleep behavior, feeling of thirst, drug sensitivity etc., as will be discussed below. While they can hardly be distinguished from others under baseline conditions, they respond differently to stimuli such as cold, mechanical or psychological stress, etc. Among the most apparent pathological reactions are the vasoconstrictions giving rise to the previously used term “vasospastic syndrome” [21]. But why do these subjects constrict their vessels more than others?

Subjects with a PVD have a normal capacity to produce ATP and are, therefore, physically seen as strong as the others. Under certain circumstances, however, where these subjects do not use as much ATP, such as when they sit quietly in a cold environment, they are not able to produce as much heat as others. This is one of the reasons why these subjects constrict the vessels in their extremities: to reduce heat loss. Interestingly, as they constrict the vessels in their skin they also constrict the vessels in the back of their eyes.

The PVD syndrome occurs more frequently in females than in males [22], in Japanese than in Caucasians [23,24], and in academics than in blue collar workers [25]. It remains unclear why women suffer more often from PVD than men. The symptoms are normally first manifested in puberty and mitigate with age.

There are various clinical signs that indicate a PVD. Subjects with a PVD often have cold extremities like cold hands or feet [26], they tend to have normal or low body mass indices [27], the feeling of thirst is often reduced (they drink because they know they have to drink and not so much because they are thirsty) [28], they tend to have low blood pressure especially when they are young [29], they more often suffer from migraines than non-PVD subjects [30] (although PVD and migraine are two distinct entities). PVD subjects have often an altered drug sensitivity due to differential expression of ATP-Binding Cassette (ABC) transporter proteins [31]. They have, on average, a longer sleep onset time, especially when they are cold [32]. They often have a meticulous personality and are often successful in their professions [33].

The regulation of OBF in patients with PVD is different: morphologically the retinal vessels demonstrate a higher level of irregularity [34]. When stimulated by flicker light they respond with a smaller vasodilation than normals [8]. In a provocation with hand-grip test (a stimulation of the sympathetic nervous system) their choroidal vessels constrict more than normals [35]. Vessels in vasospastic subjects conduct pulse waves faster and are thus stiffer than those in nonvasospastic subjects [36]. PVD subjects have a disturbed autoregulation [37] very similar to glaucoma patients that progress despite a normal or normalized IOP [38].

Dysfunction of regulation leads to an unstable ocular perfusion especially when IOP or blood pressure fluctuates. The resulting instability of blood flow leads to a repeated, but very mild, reperfusion contributing via oxidative stress to glaucomatous damage. This seems to be a key factor and will therefore be discussed below [39].

### Reperfusion injury

#### What is reperfusion injury?

Reperfusion injury refers to damage to tissue caused when blood supply returns to the tissue after a period of ischemia. The absence of oxygen and nutrients from the blood creates a condition in which the restoration of circulation results in inflammation and oxidative damage through the induction of oxidative stress rather than restoration of normal function.

In POAG, oxygen tension in the tissue often falls temporarily. This drop is very mild, but recurrent over years. To some extent, such a drop of oxygen leads to an adaptation which is called preconditioning. This makes the cell more resistant to coming drops of oxygen. If the drop of oxygen exceeds a certain limit, reperfusion damage is induced. When the oxygen drop is even larger or lasts longer tissue infarction results. This occurs rarely in glaucoma but as a consequence of other diseases.

#### Reperfusion injury inducing glaucomatous optic neuropathy (GON)

Reperfusion occurs both in patients with either high IOP or very low blood pressure exceeding the capacity of autoregulation, as well as in patients with a normal or mildly increased IOP or normal or mildly decreased blood pressure if subjects suffer from disturbed autoregulation.

**Figure 6:**
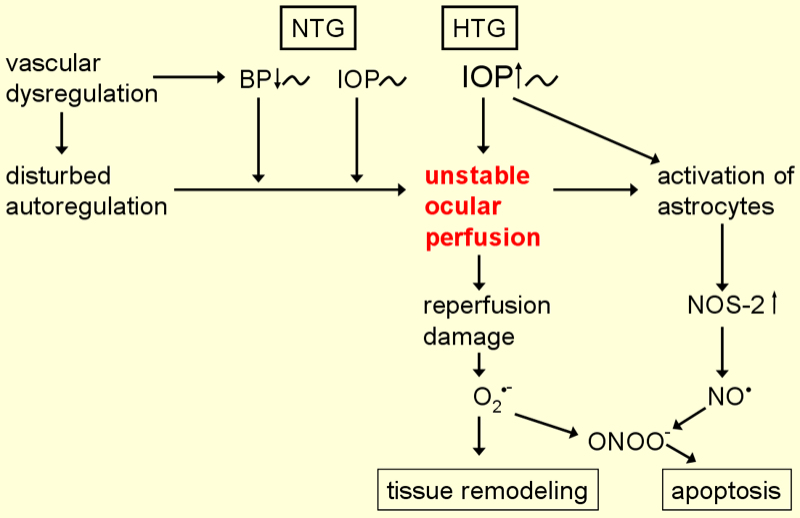
The pathogenetic concept of glaucomatous optic neuropathy. Oxidative stress results from unstable ocular blood flow (OBF). OBF is unstable both in patients with either high IOP or very low blood pressure (BP) exceeding the capacity of autoregulation, as well as in patients with a normal or mildly increased IOP or normal or mildly decreased blood pressure if subjects suffer from disturbed autoregulation. This figure is reprinted from Flammer J. Glaucoma, Glaucoma A Guide for Patients. An Introduction for Care-Providers. A Quick Reference. 3rd ed. Cambridge: Hogrefe & Huber; 2006. Figure 5.12; p 104.

Recurrent mild reperfusion leads to a chronic oxidative stress, especially in the mitochondria. Mitochondria are very crowded in the optic nerve head due to high energy consumption in the active area lacking myelin sheets. The mitochondria get more and more damaged and making energy supply less and less efficient. But also other cellular compartments suffer and undergo a sort of accelerated aging process. In parallel activated astrocytes change the microenvironment. Together a pathogenesis is induced which shall be discussed later on in this review [40] (Figure 6).

### Oxidative stress and glaucoma

#### Oxidative stress and intraocular pressure (IOP)

Oxidative stress has been implicated to be a cause of increased intraocular pressure by triggering trabecular meshwork (TM) degeneration and thus contributing to alterations in the aqueous outflow pathway [41,42]. The TM is in constant contact with the aqueous humor from which ROS may be generated through light catalyzed reactions, metabolic pathways or

inflammation [41,43-45]. Disturbance of the TM cell status by an insult such as oxidative stress, may lead to cellular loss and an overexpression or alteration in the structures of the TM, in particular in the extracellular matrix [46-48], leading to impaired aqueous humor outflow and thereby an increase in IOP.

The pathogenic role of oxidative stress in increasing IOP is supported by various studies. In vitro treatment of human TM cells with hydrogen peroxide alters cellular adhesion and integrity [46]. In an animal study of calf, perfusion of TM cells with peroxide has shown to reduce aqueous humor drainage from the anterior chamber of the eye [49]. In humans, DNA damage has been reported to be significantly higher in the TM cells of glaucoma patients than in those of age-matched controls [50]. Further studies demonstrate abundant oxidative nucleotide modification (8-OH-dG) levels in human TM to be significantly correlated to the increase in IOP and to visual field damage [49,51]. Glaucoma patients display a significant depletion of total antioxidant potential in their aqueous humor [52], a decrease in plasmatic glutathione levels [53], and an increase in serum antibodies against glutathione-S- transferase [54]. Heat shock proteins act as molecular chaperones protecting the three dimensional structure of other proteins. The heat shock protein, αB-crystalline was shown to be overly expressed in TM cells of both human and monkey eyes stressed by heat [55]. In vitro studies have shown that enhanced expression of αB-crystalline confers cellular thermoresistance [56] and protection against oxidative stress [57]. The mechanisms by which αB-crystalline performs these functions are still not clear. In glaucoma heat shock proteins are upregulated supporting indirectly the role of oxidative stress. In glaucoma, overexpression of heat shock proteins may be a consequence of oxidative stress [55].

#### Oxidative stress and glaucomatous optic neuropathy (GON)

The observation that the majority of glaucoma patients show signs of reduced ocular blood flow (OBF) as well as ischemic signs in the eye (e.g., upregulation of the ischemia inducible factor 1-α) [58], indicates that other factors, in particular hemodynamic factors are involved in the pathogenesis as well [2].

OBF is, however, also reduced in a number of other diseases as for example in multiple sclerosis [18], (due to a high level of circulating endothelin) [15], indicating that ischemia by itself does not induce GON. Even patients with a carotid stenosis do not suffer significantly more often from GON [59].

While on the one hand, glaucoma patients have reduced OBF and this OBF reduction even has a predictive power for the progression of GON [60], on the other hand, a blood flow reduction by artherosclerosis, multiple sclerosis or by a high level of endothelin-1 (e.g., multiple sclerosis), do not increase the risk for GON significantly. The only way to explain this is simply the fact that it is not a stable reduction in OBF but rather the instability in blood flow that may lead to GON [2]. Such an instablity in OBF leads to a repeated mild reperfusion injury [39]. Reperfusion injury in glaucoma patients, particularly in the optic nerve head is very mild but occurs repeatedly [39]. This hypothesis of the reperfusion injury being involved in the pathogenesis of GON is supported by the observation that IOP fluctuation is more damaging than a stable increase in IOP [61-63], and by observations that patients that progress despite a normalized IOP suffer from a disturbed autoregulation [38]. The main cause for this insufficient autoregulation, as previously mentioned, is a primary vascular dysregulation (PVD) syndrome [20].

**Figure 7:**
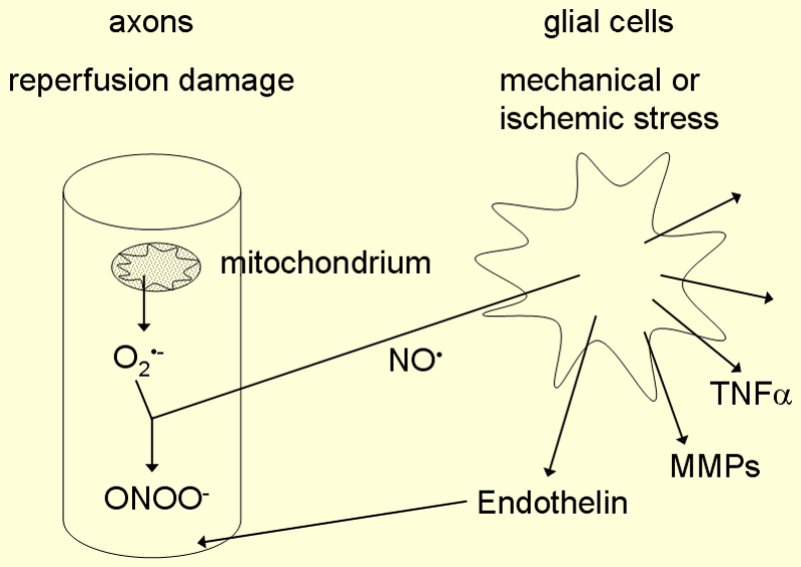
Production of peroxynitrite. The simultaneous production of the readily diffusible nitric oxide (NO) in glial cells and the superoxide anion (O2^-^) in the axons of the retinal ganglion cells leads to the production of peroxynitrite (ONOO^-^). This figure is reprinted from Flammer J. Glaucoma, Glaucoma A Guide for Patients. An Introduction for Care-Providers. A Quick Reference. 3rd ed. Cambridge: Hogrefe & Huber; 2006. Figure 5.13; p 105.

We also mentioned that recurrent mild reperfusion leads to a chronic oxidative stress, which in turn, damages all types of molecules and thereby reduces the probability of cellular survival. Still, the question arises as to why in glaucoma specifically the retinal ganglion cells and their axons die by apoptosis. Both ischemic and mechanical stress lead to an activation of astrocytes. Once activated, astrocytes start producing a variety of molecules, one of which is nitric oxide (NO). NO although having a very short half-life, has a small size and is liposoluble and can therefore readily diffuse to neighboring cells such as the axons of the optic nerve head (ONH). If simultaneously as a result of reperfusion, the concentration of superoxide (O_2_^-^) is high, the very damaging peroxynitrite is produced [64] (Figure 7). Both superoxide anions and peroxynitrite are water soluble and can therefore not diffuse out of an intact cell membrane. We hypothesize that they diffuse within the axons both towards the retina and the lateral geniculate nucleus. In fact, nitrosylation of SH groups have been found in the retina and in the lateral geniculate nucleus indicating the presence of peroxynitrite. The assumption of reperfusion injury being involved in the pathogenesis also explains why sleep apnoe [65] or reversible shock-like states [66] can lead to GON.

#### Signs of oxidative stress

Various signs indicate that oxidative stress plays an important role in the pathogenesis of GON. If we rely on the fact that oxidative stress occurs, we would expect increased number of DNA breaks, an upregulation of Endothelin-1 (ET-1), an upregulation of metalloproteinase-9 (MMP-9), and increased activity of proteosomes. This is indeed all the case as described below.

**Figure 8:**
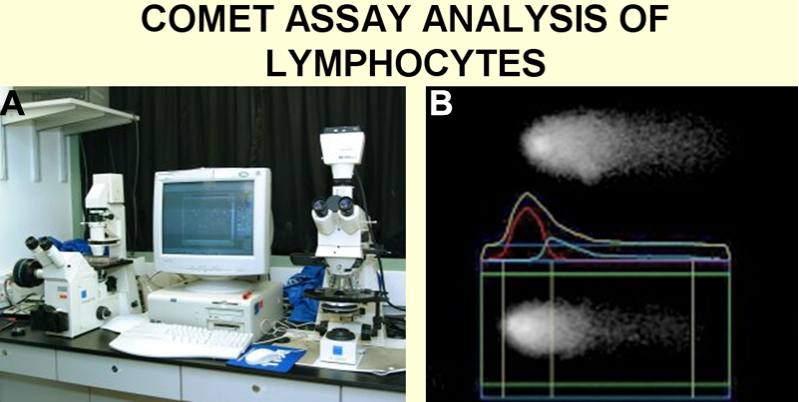
Comet assay analysis of lymphocytes. **A**: Photograph of the present equipment used. **B**: a typical picture of the DNA of a lymphocyte analyzed by the system. The greater the amount of DNA breaks, the larger the comet tail.

Analysis of DNA breaks by means of comet assay in the circulating lymphocytes revealed an increase in DNA breaks in glaucoma patients compared to age-matched controls [67] (Figure 8). The increase in DNA breaks may partially be due to an increase in oxidative stress damaging DNA but also partially due to a slow down of DNA repair. The latter view is supported by the observation that there is a reduced expression of the Xeroderma pigmentosum gene (XPGC) in glaucoma patients [68].

Oxidative stress also leads to an unspecific increase in endothelin-1 (ET-1). Various studies have demonstrated an increase in ET-1 in glaucoma subjects, in particular in patients that progress despite normalized IOP [69]. Metalloproteinases (MMP-2 and MMP-9) are upregulated in the optic nerve head of glaucoma patients. There is an upregulation of MMP-9 even in circulating lymphocytes [70]. The upregulation of MMP-9 not only fits the hypothesis of reperfusion injury, but also explain various steps in the pathogenetic process of GON. Oxidative stress damages a variety of molecules including proteins. Unlike damaged DNA, damaged proteins cannot be repaired. Nature has developed sophisticated methods to eliminate damaged intracellular proteins. These proteins are first marked by ubiquitin and then pulled electrostatically into the proteosomes where they are cut in pieces which are then recycled. The activity of the proteosomes therefore gives us an indirect measure of proteins damaged. Indeed, the expression of 20S proteosome alpha subunit is upregulated in glaucoma, further supporting the hypothesis of an oxidative stress [71].

These pro-apoptotic conditions observed in the circulating lymphocytes would theoretically lead to a reduced half life of these leukocytes. Fortunately nature has built in a protective mechanism. The genes known to induce apoptosis, namely P21^WAF1/CIP1^ and 14–3-3 sigma, were observed to be down-regulated as a result of hypermethylation of DNA [67].

### Conclusions

The exact mechanisms that lead to damage of the trabecular meshwork and thereby to an increase in IOP, in POAG are still unknown. The pathogenesis leading to GON is also only partially known. Obviously there are a number of factors and mechanisms involved. These factors are most likely interrelated, as in a “complex network.” So it not the question as to whether a glaucomatous damage is more likely due to one or the other factor (e.g., pressure versus vascular factors) but rather a question as to how these and other factors play together in an individual patient. One of these factors is ocular blood flow. The other, is oxidative stress. Unfortunately, we have still only a vague idea as to how oxidative stress is brought about in glaucoma and how it damages the tissue. The elucidation of these factors will lead to the development of new treatment strategies [72-76].
